# The Effects of Eccentric Contraction Execution Time on the Properties of the Patellar Tendon

**DOI:** 10.3390/ijerph19159296

**Published:** 2022-07-29

**Authors:** Fernando Martínez, Pablo Abián, Fernando Jiménez, Javier Abián-Vicén

**Affiliations:** 1 Performance and Sport Rehabilitation Laboratory, Faculty of Sport Sciences, University of Castilla-La Mancha, 45071 Toledo, Spain; fermasa83@gmail.com (F.M.); josefernando.jimenez@uclm.es (F.J.); 2 Faculty of Humanities and Social Sciences, Comillas Pontifical University, 28049 Madrid, Spain; pabian@comillas.edu

**Keywords:** eccentric training, decline squat, patellar tendon, sonoelastography, stiffness

## Abstract

The purpose of this study was to assess the effects of eccentric contraction execution time on the morphological and elastic properties of the patellar tendon (PT) in a six-week, single-leg decline squat (SLDS) exercise training program. In addition, the effects of a six-week detraining period on the same variables were evaluated. Fifty participants were randomized into the control group (CG; n = 15), experimental group 1 (EG6s; n = 17; eccentric contraction execution time = 6 s) and experimental group 2 (EG3s; n = 18; eccentric contraction execution time = 3 s). The thickness and elastographic index (EI) in different regions of interest (ROIs) in the PT were measured after 6 weeks of eccentric training using the single-leg decline squat exercise (three sessions per week, 80% of the eccentric one-repetition maximum) and after 6 weeks of detraining. There was an increase in the thickness of the PT in the different ROIs analyzed in both experimental groups at the end of the training period. Especially worth noting was the increase in the thickness of the PT at the proximal level in EG3s (*p* = 0.001), and the increase at the distal level in EG6s (*p* = 0.001). On the other hand, there was a reduction in EI in EG6S at the end of the intervention program (*p* = 0.021), and both experimental groups increased EI in the three regions of interest analyzed after the detraining period (*p* < 0.01). In conclusion, the execution time of the eccentric contraction in the SLDS exercise determines the anatomical level of the morphological adaptations in the PT. These morphological adaptations are lost after 6 weeks of detraining, producing an increase in tendon stiffness.

## 1. Introduction

The patellar tendon (PT) originates from the distal two-thirds of the patella and generally inserts at the most distal end of the anterior tuberosity of the tibia, where it joins the fibrillar expansions of the iliotibial tract [1]. Three vastii and part of the rectus femoris act on the knee joint by way of the vastus aponeurosis, which is likely to have a role in knee pathomecahnics [2]. Functionally, the PT is an energy storing structure that transmits muscle-derived forces that produce joint motion and stores and releases energy, which has the capability to enhance power and efficiency in extension [3,4]. The PT stores and releases a great deal of energy during explosive movements and this possibly contributes to the high incidence of patellar tendinopathy in elite athletics [5,6]. PT fibers do not have the same characteristics which can generate different adaptations to mechanical stimulation in the different sections of the tendon; Basso et al. [7] reported that the posterior fibers of the PT are exposed to the greatest strains when the quadriceps tendon is loaded with the knee flexed.

Eccentric exercise is frequently used in physiotherapeutic interventions for the treatment of patellar tendinopathy [8]. Specifically, the eccentric single-leg decline squat (SLDSe) exercise has been one of the most widely used because it offers superior results compared with other exercises [9]. Furthermore, different investigations show the ability of the tendon to morphologically and mechanically adapt its structural components to external load stimuli [10,11,12]. In this respect, it seems that high loads and longer intervention durations (>12 weeks) may be the most influential variables to produce these modifications [11,13,14].

Movement speed during eccentric training alters important factors involved in muscular adaptations, such as time under tension and muscle activation [15,16]. Burd et al. [17] found that greater muscle time under tension increased mitochondrial and sarcoplasmic protein synthesis. Fast eccentric movement during training has been found most effective for muscle hypertrophy and strength gain [16,18] and slow eccentric movement during training has had larger effects on the elastic and structural properties of the PT [16]. However, there is a need to investigate the different components of the eccentric exercise load on the morphological and mechanical properties of the muscle and tendon structures. Therefore, the aims of this study were: (1) to compare the effects of the execution time of the eccentric contraction (3 s vs. 6 s) on the morphological and elastic properties of the PT after a 6 week SLDSe training program, and (2) to analyze the changes in these variables after a 6 week detraining process.

## 2. Materials and Methods

### 2.1. Participants and General Procedure

Fifty-four healthy physical education students voluntary participated in this study. Eligible participants were physically active (3–6 h per week of moderate physical activity) and between 18 and 35 years old. All participants scored > 90 points on the Victorian Institute of Sport Assessment-PT questionnaire (VISA-P) in its version translated to Spanish (VISA-P-Sp) [19] to exclude symptoms of patellar tendinopathy, and they were required to keep their normal exercise practices throughout the study. Participants were excluded from the investigation if they: (1) had had an injury in the lower limbs during the last 2 years; (2) had performed strength training in the lower limbs during the last 8 weeks; and (3) practiced basketball or volleyball (sports where jumping is an important action) more than 2 h per week or competitively. The sample size was previously calculated based on existing research [10] which measured the influence of resistance training on PT stiffness. The minimal number of participants required to attain a power of 0.9 and a bilateral alpha level of 0.05 was calculated to be 10 participants per group.

**Figure 1:**
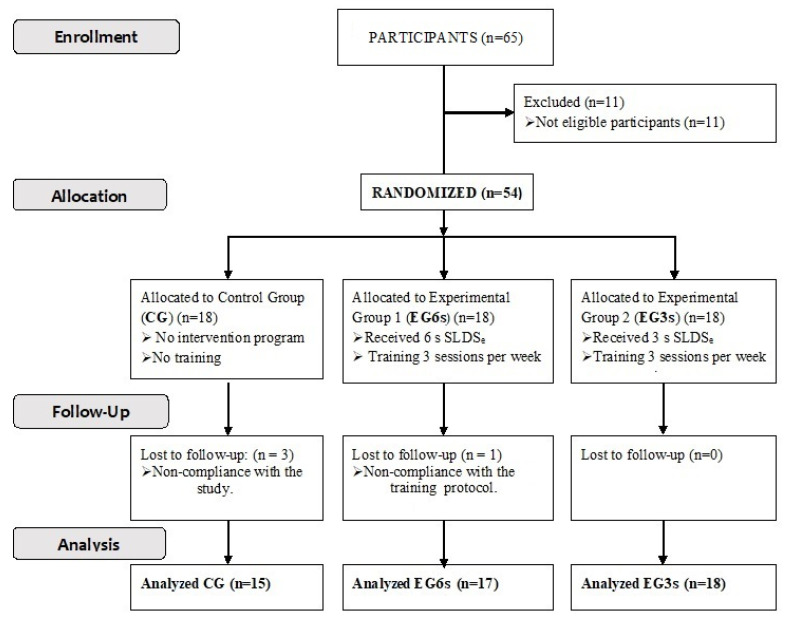
Participants and inclusion procedure.

Participants were randomly divided into three groups: (1) the control group (CG; n = 15; 21.3 ± 2.8 years; 67.22 ± 12.25 kg; 1.72 ± 0.09 m and 95.8 ± 1.9 score in the VISA-P-Sp; 3 participants were lost during the follow-up of the study) with no intervention program; (2) experimental group 1 (EG6s; n = 17; 21.2 ± 2.2 years; 67.14 ± 12.37 kg, 1.72 ± 0.09 m and 95.4 ± 2.1 score in the VISA-P-Sp; 1 participant was lost during the follow-up of the study) which followed the intervention program performing the SLDEe in 6 s; and (3) experimental group 2 (EG3s; n = 18; 21.3 ± 2.5 years; 67.68.27 ± 11.85 kg, 1.70 ± 0.10 m and 96.1 ± 2.4 score in the VISA-P-Sp) which followed the intervention program performing the SLDEe in 3 s (Figure 1).

All participants were informed about the objective and methods of the investigation and signed the informed consent forms before the start of the study. Ethical approval was obtained from the Ethics Committee of Clinical Research at the Hospital Complex in Toledo (Spain) (62-100615) according to the principles of the latest version of the Declaration of Helsinki.

### 2.2. Experimental Design

A three-group parallel repeated measures design was carried out to examine the thickness and elastography index adaptations of the PT in response to two SLDSe training programs performed with two execution times (6 s vs. 3 s). In week −1, the participants were selected and the VISA-P-Sp questionnaire was filled out. In week 0, two sessions were carried out. In session 1, the first assessment (PRE) was made before the intervention program (these data were used as baseline measures) and the anthropomorphic measures were recorded. Participants were then notified of their allocated training group and familiarized with the eccentric SLDSe exercise and with the 5 repetition maximum (RM) test of their corresponding exercise-specific training program (no training, 3 s of SLDSe or 6 s of SLDSe). In session 2 (72 h after session 1), the 5 RM test of the SLDSe exercise for EG6s and EG3s in the dominant limb was performed in order to determine the training load.

**Figure 2:**
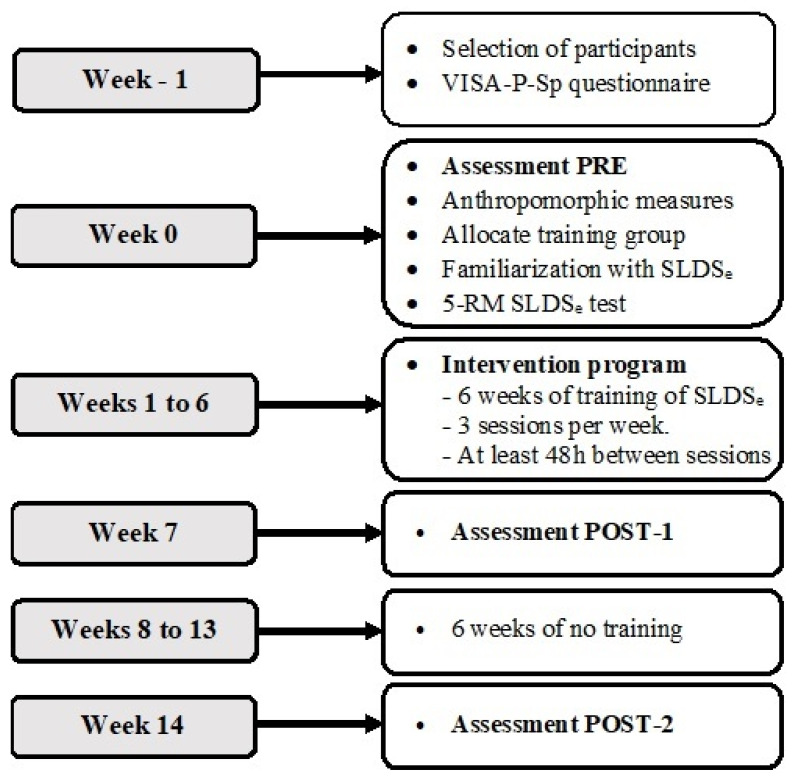
Design of the investigation.

From weeks 1 to 6 the participants completed a 6 week eccentric program. Each session was carried out with the principal investigator and two collaborators to ensure adherence and the correct technique. In week 7, the second measurement was performed at the end of the intervention program (POST-1) and in week 14, the third measurement was performed, 6 weeks after the intervention finished (POST-2) to evaluate the consequences after a 6 week detraining period (Figure 2).

### 2.3. Intervention Program

The intervention program of the SLDSe training for both groups (EG6s and EG3s) lasted 6 weeks. Three training sessions were carried out every week at least 48 h apart. Three sets of 8 repetitions were performed in each training session (the rest duration between repetitions was 6 s and between sets was 2 min) of the SLDSe exercise previously described [20] with the dominant lower limb at 80% of the 1 RM calculated previously for each group in their specific working execution time. A 25° decline board was used to perform the SLDSe in both experimental groups [20].

The eccentric phase of movement of each repetition lasted 6 s in EG6s and 3 s in EG3s. The 1 RM of the SLDSe for each experimental group was calculated before the start of the training program (in week 0) and just after the training in weeks two and four to recalculate the training load. Participants were trained to complete the back-squat exercises with the trunk in a vertical position in a multipower machine (Technogym, Gambettola, Italy). Both experimental groups were trained to perform the exercise by slowly bending the knee to 90° of flexion and, in order to focus only the eccentric loading of the quadriceps and to return to the initial position, the participants were trained to use the non-dominant lower limb in addition to the dominant one and two assistants lifted the weights to prepare them for the next repetition. Thereby, we decreased the influence of the concentric phase of the anterior thigh muscles. A metronome (www.webmetronome.com; accessed on 5 February 2019) was employed to establish the start and the end of the eccentric phase in each group.

### 2.4. 5-RM SLDSe Test

The 5 RM SLDSe test was used to calculate 1 RM. This test was performed with the dominant lower limb in a multipower system (Technogym, Gambettola, Italy), with two parallel straight bars that only allowed vertical movements. The participants were trained to position themselves under the shoulder bar of the multipower rack machine. A specific warm-up routine was carried out using the weight of the bar (~18 kg), the participants performed two sets of twelve eccentric repetitions, and one minute rest. Each of the eccentric repetitions was carried out by the two experimental groups in a time of 6 s (EG6s) or 3 s (EG3s), reaching up to 90° of knee flexion, which was assessed with a goniometer placed on the knee joint, and resting for 6 s between each repetition. After 2 min of rest, the test was carried out with the participants performing a series of 5 eccentric repetitions with increasing intensity, starting with the weight registered in the pre-testing. The load was increased until the 5 RM was determined with a 2 min rest between sets. If the subject did not keep the execution speed of the movement or did not reach 90° of knee flexion, the repetition was considered invalid. In the event that the participants could not perform the repetition due to fatigue or inability to endure the weight, the weight lifted in the last series of 5 RM was recorded. Afterwards, the 5 RM was converted to 1 RM [11,21].

### 2.5. Thickness of PT Assessment

**Figure 3:**
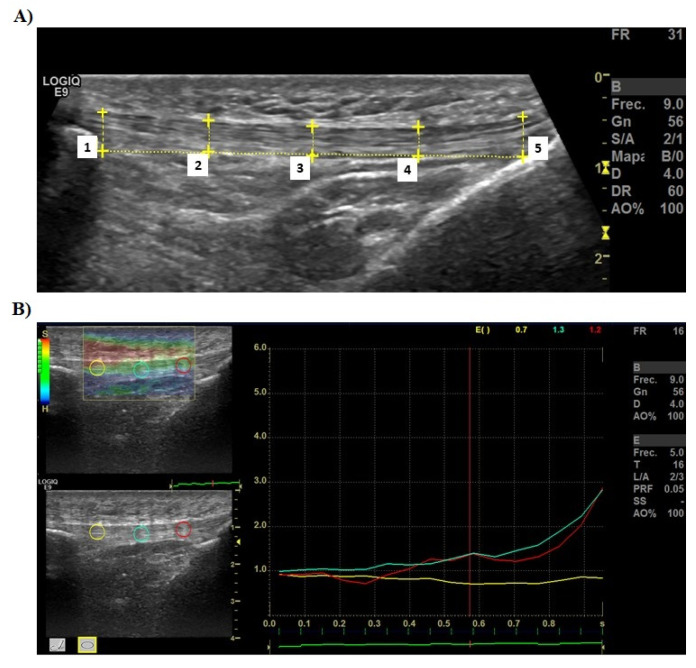
(**A**) B-mode ultrasound image of the Achilles tendon in a longitudinal plane. (**B**) Example of elastography measurement of the Achilles tendon.

The thickness of the PT was measured by B-mode ultrasonography (LOGIQ E9, GE Healthcare, Milwaukee, WI, USA) with a 5.5 cm, high frequency 6–15 MHz linear probe. The participants lay supine on a stretcher, with the knee flexed to 15° [16,22]. The transducer was placed parallel to the direction of the PT fibers. The length of the PT was measured from the lower pole of the patella to the anterior tuberosity of the tibia (deep insertions of the PT). The tendon thickness was measured in five regions of interest (ROIs) (lower pole of the patella, at 25%, 50% and 75% of the total PT length, and anterior tuberosity of the tibia) with the specific software for the US machine (Figure 3). These measurements have been used in previous research and have shown acceptable reliability [23].

### 2.6. Elastic Properties of PT Assessment

The elastographic index (EI) of the PT was evaluated in three ROIs (at 25%, at 50% and at 75% of the total length PT; Figure 3) and in the same position as in the previous measurement by real-time elastography (LOGIQ E9, GE Healthcare, Milwaukee, WI, USA). This measurement was made following the methodology of previous research [24,25,26,27,28]. The average of the three measurements was stated. The B-mode screen was set as translucent and color-coded, real-time images were used to record the sonograms. The color code showed the strain of the tissues within the ROI, in which blue indicated hard elasticity, green and yellow corresponded to medium elasticity, and red indicated soft elasticity. A lower value of EI is related to a lower stiffness level. The ultrasound evaluation was carried out by one of the authors, F.J., who has proven experience with this type of measurement. 

### 2.7. Statistical Analysis

The data were analyzed with the statistical package IBM SPSS Statistics 23.0 (SPSS, Chicago, IL, USA). The normality of each variable was initially tested with the Shapiro-Wilk test. All the variables presented normal distributions (*p* > 0.05). Then, the main effects of the two training interventions were determined by a two-factor (3 × 3) mixed-model ANOVA. The first factor was the group (CG, EG6s and EG3s) and the second factor was the timeline (PRE-, POST-1 and POST-2). Effect size (ES) statistics were calculated according to the formula proposed by Cohen [29] to quantify the magnitude of the difference in pairwise comparisons. The magnitude of Cohen’s effect size was interpreted using the following scale: an ES lower than 0.2 was considered as small, an ES around 0.5 was considered as medium and an ES over 0.8 was considered as large. All data were presented as mean ± standard deviation. The significance level was set at *p* < 0.05 for all statistical analyses. 

## 3. Results

In the PRE-tests, no outcome measures differed statistically among groups (CG, EG6s and EG3s) except between CG and EG3s in the thickness of PT at 50%. Additionally, in the CG, no significant differences were found between the measurements obtained at PRE, POST-1 and POST-2 tests in any of the dependent variables. No significant differences were found in the length of the PT between PRE, POST-1 and POST-2 evaluations in any of the groups.

A significant increase was found after 6 weeks of eccentric training in the 1 RM in both experimental groups (EG6s: difference = 89.7 ± 29.9 kg; confidence interval (CI) 95%: from 73.2 to 106.2 kg, *p* < 0.001, ES = 3.8 and EG3s: difference = 100.5 ± 33.7 kg; CI 95%: from 84.5 to 116.5 kg, *p* < 0.001, ES = 3.9) and a decrease after 6 weeks of detraining was found in EG6s (difference = −30.8 ± 24.2 kg; confidence interval (CI) 95%: from −43.0 to −18.6 kg, *p* < 0.001, ES = 0.8) and EG3s (difference = −41.9 ± 21.0 kg; CI 95%: from −53.8 to −30.1 kg, *p* < 0.001, ES = 1.0). No significant changes in the 1 RM were found in the control group.

The values obtained for the length and the thickness of the PT in the different zones analyzed are shown in Table 1. The thickness of the PT in the lower pole of the patella was 0.03 ± 0.03 cm (CI 95%, from 0.01 to 0.06 cm, *p* = 0.015, ES = 0.4) greater in EG3s after 6 weeks of eccentric training and a decrease after a 6 week follow-up was found in EG3s (*p* = 0.005). A significant increase was found after 6 weeks of eccentric training in the thickness of the PT at 25% in both experimental groups (EG6s: difference = 0.05 ± 0.02 cm; CI 95%: from 0.03 to 0.06 cm, *p* < 0.001, ES = 0.6 and EG3s: difference = 0.03 ± 0.03 cm; CI 95%: from 0.01 to 0.04 cm, *p* = 0.001, ES = 0.4) and a decrease after 6 weeks of detraining was found in EG6s (*p* < 0.001) and EG3s (*p* < 0.001). The values in the thickness of the PT at 50% and 75% were greater in EG6s (*p* < 0.001) and EG3s (*p* = 0.013 and *p* = 0.001, respectively) after 6 weeks of eccentric training. Significant decreases (*p* < 0.05) were found after the six weeks of non-training compared to POST-1 in the thickness of the PT at 50% (EG6s: *p* < 0.001) and 75% (EG6s: *p* < 0.001; and EG3s: *p* = 0.024).

The thickness of the PT in the anterior tuberosity of the tibia in EG6s was 0.05 ± 0.04 cm (CI 95%, from 0.02 to 0.07 cm, *p* < 0.001, ES = 0.5) greater after the 6 weeks of eccentric training compared to the baseline and a decrease from POST-1 was found after 6 weeks of detraining (EG6s: difference = 0.08 ± 0.07 cm; CI 95%: from 0.04 to 0.12 cm, *p* < 0.001, ES = 0.9). 

**Figure 4:**
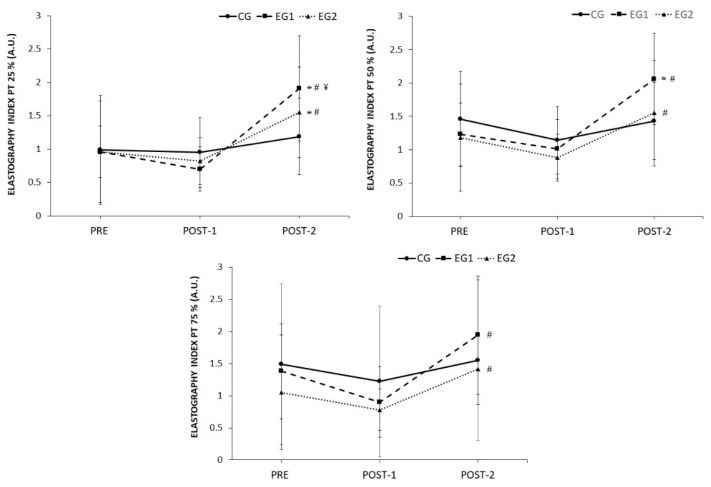
Elastic properties of the patellar tendon (PT) at 25%, 50% and 75% of the length of the PT after six weeks of eccentric training (POST-1) with two execution times (EG6s = 6 s and EG3s = 3 s) and after a 6 week follow-up of detraining (POST-2). ¥ = *p* < 0.05 from CG; # = *p* < 0.05 from POST-1 evaluation; * = *p* < 0.05 from pre-evaluation.

Significant increases (*p* < 0.05) in the EI at 25% of the PT were found after the six weeks of non-training compared to PRE evaluation (EG6s: *p* = 0.002; and EG3s: *p* = 0.039) and compared to POST-1 (EG6s: difference = 1.21 ± 0.77 A.U.; CI 95%: from 0.72 to 1.69 A.U., *p* < 0.001, ES = 2.2; and EG3s: difference = 0.72 ± 0.81 A.U.; CI 95%: from 0.30 to 1.15 A.U., *p* < 0.001, ES = 1.4). A significant increase (*p* < 0.05) was found after 6 weeks of detraining compared to POST-1 in the EI at 50% of the PT (EG6s: *p* < 0.001; and EG3s: *p* = 0.001) and in the EI at 75% of the PT (EG6s: difference = 1.04 ± 1.08 A.U.; CI 95%: from 0.50 to 1.57 A.U., *p* < 0.001, ES = 1.4; and EG3s: difference = 0.64 ± 0.52 A.U.; CI 95%: from 0.17 to 1.10 A.U., *p* < 0.001, ES = 1.5) in both experimental groups. In the same way, EG6s showed an increase (*p* < 0.05) in POST-2 from PRE evaluation in the EI at 50% of the PT (Figure 4).

## 4. Discussion

The main purpose of this study was to compare the effects of the execution time of eccentric contraction (3 s vs. 6 s) on the morphological and elastic properties of the PT after a 6 week SLDSe training program. Although changes in the thickness of the PT were evidenced in both experimental groups after the completion of the SLDSe program, it is worth noting an increase of ~7% at the level of the lower pole of the patella in EG3s, and an increase of ~10% at the level of the anterior tuberosity of the tibia in EG6s. This is an important finding to be taken into account by physical therapists and sports adapters in the recovery of patellar tendinopathy, since depending on the level of the injury in the PT (proximal or distal), one execution time of the eccentric contraction or the other should be used in the SLDSe exercise.

Although the tendon is considered a poorly vascularized structure [30], it has been shown to respond to external mechanical loads by altering its biomechanical (Young’s modulus) [31,32] and/or morphological (CSA) [10,33] properties. These responses depend fundamentally on the activity and intensity of the stimulus to which it is subjected [34]. To our knowledge, this is the first study that has compared the execution time effects of an eccentric contraction in SLDSe exercise on the morphological and elastic properties in different regions (lower pole of the patella, at 25%, 50% and 75% of the total PT length, and anterior tuberosity of the tibia) of healthy PT. Previous research shows that the areas of greatest increase in PT CSA are located at the proximal and distal ends [10,33,35]. This may be due to the fact that they are the areas in which the tendon is most vascularized [10]. In this respect, the differences found between both experimental groups in the ROIs analyzed may be due to the time during which the PT was subjected to stress [36]. Although participants were urged at all times to maintain a constant speed in the eccentric contraction, it is possible that longer execution times would cause the participant to spend more time with the degree of knee flexion close to 90°, thus transferring more tension to the anterior tuberosity of the tibia zone. Conversely, shorter execution times could have produced the opposite effect, and put the PT under stress for a longer time in degrees closer to full extension. A solution to this limitation of the study could be in the placement of a digital goniometer on the knee joint that controls the instantaneous angular speed in the path of the execution of the eccentric contraction to test this hypothesis.

Previous studies in animal models have shown that eccentric exercise modifies tendon gene expression producing an increase in collagen synthesis [37,38]. In the case of human tendons, it has been shown that high loads can increase collagen synthesis 48–72 h after exercise [39], but in the bibliographic review carried out to date, studies were not found that specifically evaluate the effect of execution time of the eccentric contraction on collagen expression, its growth factors and the ability to produce hypertrophy in human tendons.

The results obtained in tendon thickness agree with previous investigations that have analyzed the thicknesses of healthy and pathological tendons [16,40]. On the other hand, although most of the investigations that have achieved hypertrophic increases in the PT have used at least twelve weeks of intervention by means of eccentric exercise [10,11,33,35], the present study, according with Abian et al. [16], shows that six weeks of intervention using the SLDSe exercise focused on its eccentric phase are sufficient to increase the thickness of the PT, independently of the execution time of the eccentric contraction performed. This may be due to the high load (80% of the eccentric 1 RM) and the type of exercise (SLDSe) used in our intervention program.

No research has been found in the literature that has evaluated the residual effects on the morphological properties of the PT after the cessation of a training program using eccentric contractions. In the present investigation, it was observed that after hypertrophy in the tendon at the end of the intervention, the tendons of both experimental groups returned to their previous values recorded at the beginning of the study six weeks after cessation of activity. This situation may be due to the fact that, in addition to an increase in the synthesis of the collagen process, there is an increase in the concentrations of internal water and additional material that are more easily lost after the cessation of activity [41].

In 2001, an increase in PT stiffness was reported for the first time after twelve weeks of training using isometric contractions [42]. Moreover, sonoelastography has proven to be a reliable method in the exploration of the EI of healthy PTs [43]. In the present investigation, no differences were observed at the end of the intervention, and increases were found in both experimental groups (EG3s and EG6s) after the detraining period. Few studies were found in the literature review showing a decrease in tendon stiffness after an intervention program using eccentric contractions [12,13]. The results in these studies found a decrease of 6–15%. It should be noted that none of the previous investigations used sonolastography for evaluating the PT stiffness index or the detraining period, which makes it difficult to compare with the data recorded in this study. 

On the other hand, transient reductions in the hydration of Achilles [44] and PTs [45] have been found after undergoing physical exercise. Further research is required to ascertain if the reduction in EI observed in EG6s after the end of the intervention period is associated with a change in the hydration status of the PT or with the realignment of the newly formed collagen fibers. In our research it was observed that six weeks after the end of the intervention, both experimental groups increased the EI of the PT in the ROIs evaluated. Other previous investigations found a reduction in the stiffness index of Achilles [46] and PTs [47] one month and two months, respectively, after completing a training program using isometric contractions. These discrepancies in the results obtained may be due to the different evaluation methodologies used and the characteristics of the intervention program, with eccentric exercise being able to cause adaptations in the stiffness index over a longer term than isometric exercise. Furthermore, the increase in EI coupled with the reduction in PT thickness found after the detraining process may be due to a change in the mechanical properties of the components that make up the tendon structure. These properties may have been modified through mechanisms, such as increased packaging of collagen fibers or through alterations in the angle projected by them [48].

The PT is considered to be a short and thick tendon whose main function is to transmit the forces generated in the quadriceps to the tibia. In addition, the PT has other important functions, such as energy storage/release in the articular loading and unloading phases and protection against muscle injuries [49]. Therefore, the increase in the stiffness of the PT found in our research could be adequate to transmit the forces quickly and efficiently, but it could affect its mechanical damping function and its elastic energy-saving capacity for economy of movement [40].

## 5. Conclusions

In conclusion, 6 weeks of SLDSe produced significant changes in the morphological properties of the PT. These adaptations were lost after 6 weeks of detraining. It was observed that longer times of execution of the eccentric contraction were associated with increases in the thickness of the PT at the distal level, and shorter times with increases at the proximal level. On the other hand, longer times of execution of the eccentric contraction induced a reduction in the tendon EI, and that regardless of the time of execution of the eccentric contraction, after 6 weeks of detraining there was an increase in the stiffness of the PT.

## Figures and Tables

**Table 1:** Morphological properties of the PT in response to six weeks of eccentric training (POST-1) with two execution runtimes (EG6s = 6 s and EG3s = 3 s) and a 6 week follow up of detraining (POST-2).

	PRE	POST-1	POST-2	ANOVA Main Effects	F	*p*-Value
Length PT (cm)						
CG	4.53 ± 0.42	4.45 ± 0.47	4.44 ± 0.46	Time	0.37	0.694
EG6s	4.49 ± 0.64	4.45 ± 0.58	4.46 ± 0.55	Group	0.36	0.702
EG3s	4.47 ± 0.43	4.38 ± 0.39	4.47 ± 0.38	Time × group	0.60	0.667
Thickness PT in patella (cm)						
CG	0.48 ± 0.05	0.48 ± 0.06	0.47 ± 0.05	Time	12.35	<0.001
EG6s	0.45 ± 0.10	0.47 ± 0.09	0.43 ± 0.08 #	Group	0.48	0.621
EG3s	0.45 ± 0.08	0.48 ± 0.09 *	0.44 ± 0.07 #	Time × group	2.69	0.037
Thickness PT 25% (cm)						
CG	0.41 ± 0.06	0.40 ± 0.06	0.39 ± 0.06	Time	27.67	<0.001
EG6s	0.38 ± 0.07	0.42 ± 0.07 *	0.36 ± 0.07 #	Group	0.26	0.773
EG3s	0.39 ± 0.07	0.41 ± 0.08 *	0.35 ± 0.05 #	Time × group	8.10	<0.001
Thickness PT 50% (cm)						
CG	0.39 ± 0.06	0.37 ± 0.05	0.37 ± 0.04	Time	13.17	<0.001
EG6s	0.35 ± 0.05	0.42 ± 0.07 *	0.34 ± 0.06 #	Group	3.58	0.037
EG3s	0.31 ± 0.07 ¥	0.34 ± 0.07 *,†	0.33 ± 0.07	Time × group	7.80	<0.001
Thickness PT 75% (cm)						
CG	0.40 ± 0.05	0.39 ± 0.04	0.40 ± 0.04	Time	11.89	<0.001
EG6s	0.38 ± 0.06	0.44 ± 0.07 *	0.37 ± 0.07 #	Group	1.51	0.232
EG3s	0.36 ± 0.07	0.39 ± 0.07 *	0.35 ± 0.07 #	Time × group	3.91	0.006
Thickness PT in tibia(cm)						
CG	0.51 ± 0.07	0.49 ± 0.08	0.50 ± 0.07	Time	6.23	0.004
EG6s	0.51 ± 0.09	0.56 ± 0.10 *	0.48 ± 0.08 #	Group	1.20	0.310
EG3s	0.46 ± 0.10	0.48 ± 0.09	0.47 ± 0.07	Time × group	7.14	<0.001

¥ = *p* < 0.05 from CG; # = *p* < 0.05 from POST-1 evaluation; * = *p* < 0.05 from pre-evaluation; † = *p* < 0.05 from EG6s; PT = Patellar Tendon.

## Data Availability

The data presented in this study are available on request from the corresponding author. The data are not publicly available due to restrictions of the subjects’ agreement.
